# Redundancy in electronic health record corpora: analysis, impact on text mining performance and mitigation strategies

**DOI:** 10.1186/1471-2105-14-10

**Published:** 2013-01-16

**Authors:** Raphael Cohen, Michael Elhadad, Noémie Elhadad

**Affiliations:** 1 Department of Computer Science, Ben-Gurion University in the Negev, Beer-Sheva, Israel; 2 Department of Biomedical Informatics, Columbia University, New York, NY, USA

## Abstract

**Background:**

The increasing availability of Electronic Health Record (EHR) data and specifically free-text patient notes presents opportunities for phenotype extraction. Text-mining methods in particular can help disease modeling by mapping named-entities mentions to terminologies and clustering semantically related terms. EHR corpora, however, exhibit specific statistical and linguistic characteristics when compared with corpora in the biomedical literature domain. We focus on copy-and-paste redundancy: clinicians typically copy and paste information from previous notes when documenting a current patient encounter. Thus, within a longitudinal patient record, one expects to observe heavy redundancy. In this paper, we ask three research questions: (i) How can redundancy be quantified in large-scale text corpora? (ii) Conventional wisdom is that larger corpora yield better results in text mining. But how does the observed EHR redundancy affect text mining? Does such redundancy introduce a bias that distorts learned models? Or does the redundancy introduce benefits by highlighting stable and important subsets of the corpus? (iii) How can one mitigate the impact of redundancy on text mining?

**Results:**

We analyze a large-scale EHR corpus and quantify redundancy both in terms of word and semantic concept repetition. We observe redundancy levels of about 30% and non-standard distribution of both words and concepts. We measure the impact of redundancy on two standard text-mining applications: collocation identification and topic modeling. We compare the results of these methods on synthetic data with controlled levels of redundancy and observe significant performance variation. Finally, we compare two mitigation strategies to avoid redundancy-induced bias: (i) a baseline strategy, keeping only the last note for each patient in the corpus; (ii) removing redundant notes with an efficient fingerprinting-based algorithm. ^a^For text mining, preprocessing the EHR corpus with fingerprinting yields significantly better results.

**Conclusions:**

Before applying text-mining techniques, one must pay careful attention to the structure of the analyzed corpora. While the importance of data cleaning has been known for low-level text characteristics (e.g., encoding and spelling), high-level and difficult-to-quantify corpus characteristics, such as naturally occurring redundancy, can also hurt text mining. Fingerprinting enables text-mining techniques to leverage available data in the EHR corpus, while avoiding the bias introduced by redundancy.

## Background

The Electronic Health Record (EHR) contains valuable information entered by clinicians. Besides its immediate clinical use at the point of care, the EHR, when treated as a repository of medical information across many patients, provides rich data waiting to be analyzed and mined for clinical discovery. Patient notes, in particular, convey an abundance of information about the patient’s medical history and treatments, as well as signs and symptoms, which, often, are not captured in the structured part of the EHR. The information in notes can be found in the form of narrative and semi-structured format through lists or templates with free-text fields. As such, much research has been devoted to parsing and information extraction of clinical notes [1-3] with the goal of improving both health care and clinical research.

Two promising areas of research in mining the EHR concern phenotype extraction, or more generally the modeling of disease based on clinical documentation [4-6] and drug-related discovery [7,8]. With these goals in mind, one might want to identify concepts that are associated by looking for frequently co-occurring pairs of concepts or phrases in patient notes, or cluster concepts across patients to identify latent variables corresponding to clinical models. In these types of scenarios, standard text-mining methods can be applied to large-scale corpora of patient notes. Collocation discovery can help identify lexical variants of medical concepts that are specific to the genre of clinical notes and are not covered by existing terminologies. Topic modeling, another text-mining technique, can help cluster terms often mentioned in the same documents across many patients. This technique can bring us one step closer to identifying a set of terms representative of a particular condition, be it symptoms, drugs, comorbidities or even lexical variants of a given condition.

EHR corpora, however, exhibit specific characteristics when compared with corpora in the biomedical literature domain or the general English domain. This paper is concerned with the inherent characteristics of corpora composed of longitudinal records in particular and their impact on text-mining techniques. Each patient is represented by a set of notes. There is a wide variation in the number of notes per patient, either because of their health status, or because some patients go to different health providers while others have all their visits in the same institution. Furthermore, clinicians typically copy and paste information from previous notes when documenting a current patient encounter. As a consequence, for a given longitudinal patient record, one expects to observe heavy redundancy. In this paper, we ask three research questions: (i) how can redundancy be quantified in large-scale text corpora? (ii) Conventional wisdom is that larger corpora yield better results in text mining. But how does the observed text redundancy in EHR affect text mining? Does the observed redundancy introduce a bias that distorts learned models? Or does the redundancy introduce benefits by highlighting stable and important subsets of the corpus? (iii) How can one mitigate the impact of redundancy on text mining?

Before presenting results of our experiments and methods, we first review previous work in assessing redundancy in the EHR, two standard text-mining techniques of interest for data-driven disease modeling, and current work in how to mitigate presence of information redundancy.

### Redundancy in the EHR

Along with the advent of EHR comes the ability to copy and paste from one note to another. While this functionality has definite benefits for clinicians, among them more efficient documentation, it has been noted that it might impact the quality of documentation as well as introduce errors in the documentation process [9-13].

Wrenn *et al.*[14] examined 1,670 patient notes of four types (resident sign-out note, progress note, admission note and discharge note) and assessed the amount of redundancy in these notes through time. Redundancy was defined through alignment of information in notes at the line level, using the Levenshtein edit distance. They showed redundancy of 78% within sign-out notes and 54% within progress notes of the same patient. Admission notes showed a redundancy of 30% compared to the progress, discharge and sign-out notes of the same patient. More recently, Zhang *et al*. [15] experimented with different metrics to assess redundancy in outpatient notes. They analyzed a corpus of notes from 178 patients. They confirm that in outpatient notes, like for inpatient notes, there is a large amount of redundancy.

Different metrics for quantifying redundancy exist for text. Sequence alignment methods such as the one proposed by Zhang *et al*. [15] are accurate yet expensive due to high complexity of string alignment even when optimized. Less stringent metrics include: amount of shared words, amount of shared concepts or amount of overlapping bi-grams [16]. While these methods have been shown to identify semantic similarity of texts, they do not specifically capture instances of copy-paste operations, which reproduce whole paragraphs.

BLAST [17], the most popular sequence similarity algorithm in bioinformatics, is based on hashing of short sub-strings within the genetic sequence and then using the slower optimized dynamic programming alignment for sequences found to share enough sub-sequences.

The algorithm we present in this paper for building a sub-corpus with reduced redundancy is based on a finger-printing method similar to BLAST. We show that this algorithm does not require the slower alignment stage of BLAST and that it accurately identifies instances of copy-paste operations.

### Text mining techniques

We review two established text-mining techniques: collocation identification and topic modeling. Both techniques have been used in many different domains and do not require any supervision. They both rely on patterns of co-occurrence of words.

Collocations are word sequences that co-occur more often than expected by chance. Collocations, such as *“heart attack”* and *“mineral water,”* carry more information than the individual words comprising them. Extraction of collocation is a basic NLP method [18] and is particularly useful for extracting salient phrases in a corpus. The NSP package we use in our experiments is widely used for collocation and n-gram extraction in the clinical domain [19-22].

Collocations in a corpus of clinical notes are prime candidates to be mapped to meaningful phenotypes [19-21]. Collocations can also help uncover multi-word terms that are not covered by medical terminologies. For instance, the phrase “hip rplc” is a common phrase used to refer to the *hip replacement* procedure, which does not match any concept on its own in the UMLS. When gathering counts or co-occurrence patterns for association studies with the goal of high-level applications, like detection of adverse drug events or disease modeling, augmenting existing terminologies with such collocations can be beneficial.

Collocations and n-grams are also used for various NLP applications such as domain adaptation of syntactic parsers [23], translation of medical summaries [24], semantic classification [25]or automatically labeling topics extracted using topic modeling [26].

State of the art articles (as cited above) and libraries (such as the NSP package) do not include any form of redundancy control or noise reduction. Redundancy mitigation is currently not a standard practice within the field of collocation extraction.

Topic modeling aims to identify common topics of discussion in a collection of documents (in our case, patient notes). Latent Dirichlet Allocation (LDA), introduced by Blei *et al*. [27], is an unsupervised generative probabilistic graphical model for topic modeling. Documents are represented as random mixtures over latent topics, where each topic is characterized by a distribution over words. The words in a document are generated one after the other by repeatedly sampling a topic according to the topic distribution and selecting a word given the chosen topic. As such, the LDA topics group words that tend to co-occur. From the viewpoint of disease modeling, LDA topics are an attractive data modeling and corpus exploration tool. As illustrative examples, we show the top-20 tokens corresponding to three topics acquired from a corpus of patient notes in Table 1. The corpus consists of records of patients with chronic kidney disease.

**Table 1:** Topics extracted from our corpus using a plain LDA model


**Topic 1**	renal	ckd	cr	kidney	appt	lasix	disease	anemia	pth	iv
**Topic 2**	htn	lisinopril	hctz	bp	lipitor	asa	date	amlodipine	ldl	hpl
**Topic 3**	pulm	pulmonary	ct	chest	copd	lung	pfts	sob	cough	pna

Topic modeling has been leveraged in a wide range of text-based applications, including document classification, summarization and search [27]. In the clinical domain, Arnold *et al.*[28] used LDA for comparing patient notes based on topics. A topic model was learned for different cohorts, with the number of topics derived experimentally based on log-likelihood fit of the created model to a test set. To improve results, only UMLS terms were used as words. More recently, Perotte *et al.* leveraged topic models in a supervised framework for the task of assigning ICD-9 codes to discharge summaries [29]. There, the input consisted of the words in the discharge summaries and the hierarchy of ICD-9 codes. Bisgin *et al.*[30] applied LDA topic modeling to FDA drug side effects labels, their results demonstrated that the acquired topics properly clustered drugs by safety concerns and therapeutic uses.

As observed for the field of collocation extraction, redundancy mitigation is not mentioned as standard practice in the case of topic modeling.

### Impact of corpus characteristics and redundancy on mining techniques

Conventional wisdom is that larger corpora yield better results in text mining. In fact, it is well established empirically that larger datasets yield more accurate models of text processing (see for example, [31-34]). Naturally the corpus must be controlled so that all texts come from a similar domain and genre. Many studies have indeed shown that cross-domain learned corpora yield poor language models [35]. The field of *domain adaptation* attempts to compensate for the poor quality of cross-domain data, by adding carefully picked text from other domains [36,37] or other statistical mitigation techniques. In the field of machine translation, for instance, Moore and Lewis [38] suggested for the task of obtaining an in-domain n-gram model, choosing only a subset of documents from the general corpora based on the domain's n-gram model can improve language model while trained on less data.

In this paper, we address the opposite problem: our original corpus is large, but it does not represent a natural sample of texts because of the way it was constructed. High redundancy and copy-and-paste operations in the notes create a biased sample of the “patient note” genre. From a practical perspective, redundant data in a corpus lead to waste of CPU time in corpus analysis and waste of I/O and storage space especially in long pipelines, where each stage of data processing yields an enriched set of the data.

Downey *et al.*[39] suggested a model for unsupervised information extraction which takes redundancy into account when extracting information from the web. They showed that the popular information extraction method, Pointwise Mutual Information (PMI), is less accurate by an order of magnitude compared to a method with redundancy handling. They present a model for unsupervised information extraction which takes redundancy into account when extracting information from the web.

Methods for identifying redundancy in large string-based databases exist in both bioinformatics and plagiarism detection [40-42]. A similar problem has been addressed in the creation of sequence databases for bioinformatics: Holm and Sander [43] advocated the creation of non-redundant protein sequence databases and suggested that databases limit the level of redundancy. Redundancy avoidance results in smaller size, reduced CPU and improved annotation consistency. Pfam [44] is a non-redundant protein sequence database manually built using representatives from each protein family. This database is used for construction of Hidden-Markov-Model classifiers widely used in Bioinformatics.

When constructing a corpus of patient notes for statistical purposes, we encounter patients with many records. High redundancy in those documents may skew statistical methods applied to the corpus. This phenomenon also hampers the use of machine learning methods by preventing a good division of the data to non-overlapping test and train sets. In the clinical realm, redundancy of information has been noted and its impact on clinical practice is discussed, but there has not been any work on the impact of redundancy in the EHR from a data mining perspective, nor any solution suggested for how to mitigate the impact of within-patient information redundancy within an EHR-mining framework.

## Results and discussion

### Quantifying redundancy in a large-scale EHR corpus

#### Word sequence redundancy at the patient level

The first task we address is to define metrics to measure the level of redundancy in a text corpus. Redundancy across two documents may be measured in different manners: shared words, shared concepts or overlapping word sequences. The most stringent method examines word sequences, and allows for some variation in the sequences (missing or changed words). For example the two sentences: “*Pt developed abd pain and acute cholecystitis*” and “*Pt developed acute abd pain and cholecystitis*” would score 100% identity on shared words but only 73% identity of sequence alignment.

Our EHR corpus can be organized by patient identifier. We can, therefore, quantify the amount of redundancy within a patient record. On average, our corpus contains 14 notes per patient, with standard deviation of 16, minimum of 1 and 167 maximum notes per patient. There are also several note types in the patient record such as imaging reports or admission notes. We expect redundancy to be high across notes of the same patient and low across notes of distinct patients. Furthermore, within a single patient record, we expect heavy redundancy across notes from the same note types. We report redundancy on same patient / similar note type (we focus on the most informative note types: primary provider, follow up and clinical notes; in this analysis we ignore the template-based note types which are redundant by construction).

Within this scope, we observe in our corpus average sequence redundancy (*i.e.,* the percentage of alignment of two documents) of 29%: that is, on average one third the words of any informative note from a given patient are aligned with a similar sequence of words in another informative note from the same patient. In contrast, the figure drops to an average of 2.9% (with maximum of 8% and standard deviation of 0.6%) when comparing the same note types across two distinct patients.

The results of high redundancy in patient notes are consistent with Wrenn *et al.*[14] observations on a similar EHR dataset. The contrast between same-patient and across-patient redundancy, however, is surprising given that the whole corpus is sampled from a population with at least one shared chronic condition. Our interpretation is that the observed redundancy is most likely **not** due to clinical content but to the process of copy and paste.

**Figure 1:**
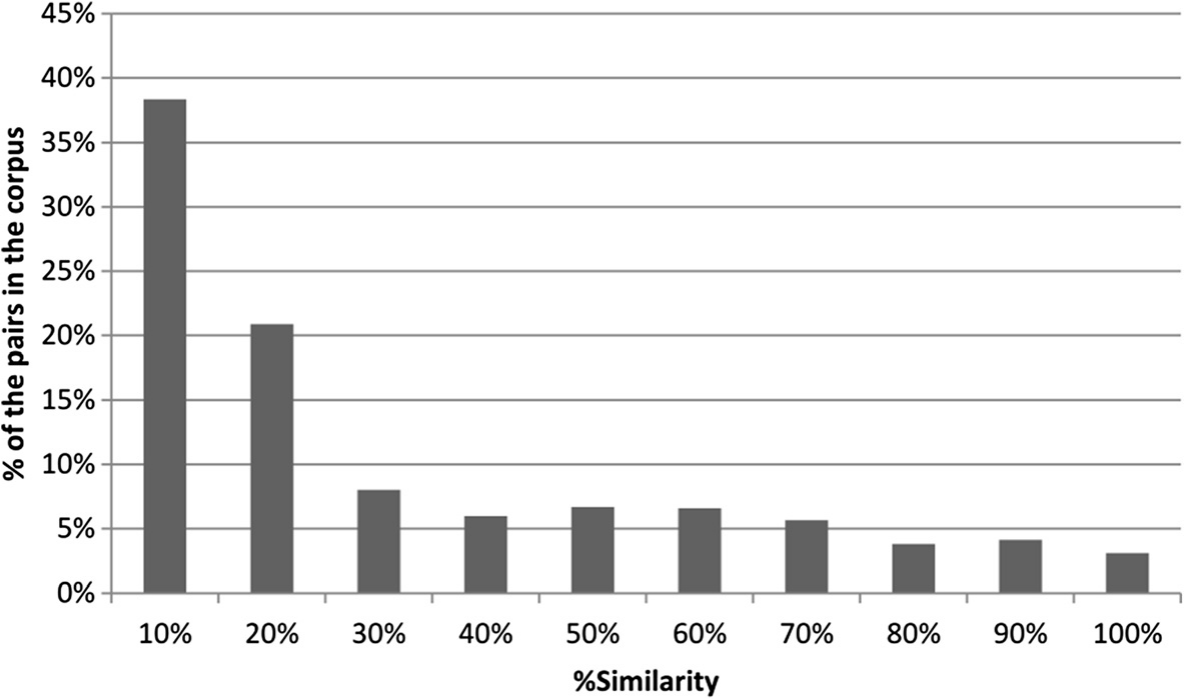
Distribution of similarity levels across pairs of same-patient informative notes in the corpus.

Figure 1 further details the full histogram of redundancy for pairs of same-patient informative notes. The redundancy (percentage of aligned tokens) was computed for the notes of a random sample of 100 patients. For instance, it indicates that 7.6% of the same patient note pairs in the corpus have between 20% and 30% identity.

The detailed distribution supports the distinction into 2 groups of notes: those with heavy repetition (about 37% of the pairs - with similarity between 40% and 100%) and those with no repetition (about 63% of the notes). A possible interpretation is that a group of patient files include many notes and tend to exhibit heavy redundancy while others are shorter with less natural redundancy. The level of overall redundancy is significant and spread over many documents (over a third).

#### Concept redundancy at the corpus level

Since free-text notes exhibit high level of variability in their language, the redundancy measures may be different when we examine terms normalized against a standard terminology. We now focus on the pre-processed EHR corpus, where named entities are mapped to UMLS Concept Unique Identifiers (CUIs) (Section 4.1.1 describes the automatic mapping method we used). We investigate whether a redundant corpus exhibits a different distribution of concepts than a less redundant one.

We expect that different subsets of the EHR corpus exhibit different levels of redundancy. The *All Informative Notes* corpus, which contains several notes per patient, but only the ones of types: “primary-provider”, “clinical-note” and “follow-up-note”, is assumed to be highly redundant, since it is homogeneous in style and clinical content. By contrast, *The Last Informative Note* corpus, which contains only the most recent note per patient, is hypothesized to be the least redundant corpus. The All EHR corpus, which contains all notes of all types, fits between these two extremes, since we expect less redundancy across note types, even for a single patient.

**Figure 2:**
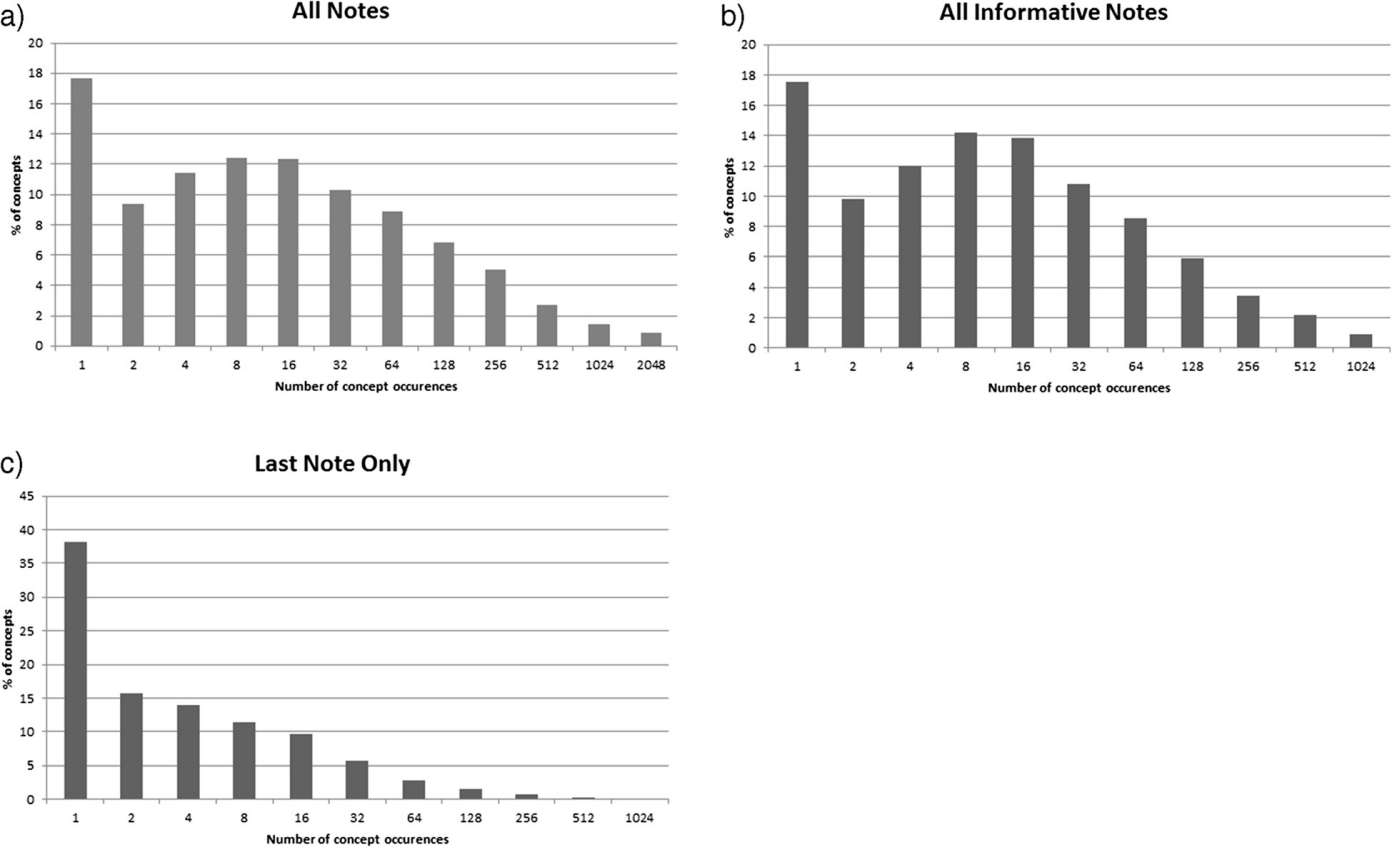
**Concept-distribution.** Distribution of UMLS concept occurrences in corpora with different levels of redundancy. The *All Notes* (**a**) and *All Informative Notes* (**b**) corpora have inherent redundancy, while the *Last Informative Note* (**c**) corpus does not. The shapes of the distributions of concepts differ depending on the presence of redundancy in the corpus.

One standard way of characterizing large corpora is to plot the histogram of terms and their raw frequencies in the corpus. According to Zipf’s law, the frequency of a word is inversely proportional to its rank in the frequency table across the corpus, that is, term frequencies follow a power law. Figure 2 shows the distribution of UMLS concepts (CUI) frequencies in the three corpora with expected decreasing levels of redundancy: the *All Informative Notes* corpus, the *All Notes* corpus, and the *Last Informative Note* Corpus. We observe that the profile in the non-redundant *Last Informative Note* corpus differs markedly from the ones of the redundant corpora (*All Notes* and *All Informative Notes*). The non-redundant corpus follows a traditional power law [45], while the redundant ones exhibit a secondary frequency peak for concepts which appear between 4 and 16 times in the corpus. In the highly-redundant *All Informative Notes* corpus, the peak is the most pronounced, with more concepts occurring four to eight times in the corpus than once.

The difference in shapes of distributions confirms in a qualitative fashion our hypothesis about the three corpora and their varying levels of redundancy. The observed contrast in distribution profiles indicates that more concepts are repeated more often than expected in the redundant corpora, and gives us a first clue that statistical metrics that rely on the regular long-tailed, power-like distributions will show bias when applied on the redundant EHR corpus. A similar pattern is observed at the bi-gram level (a Zipfian distribution for the non-redundant corpus and a non-Zipfian distribution for the redundant corpus).

### Impact of redundancy on text mining

We have observed that redundant corpora exhibit different statistical profiles than non-redundant ones, according to their word occurrence distributions. We now investigate whether these differences impact the performance of standard text mining techniques: collocation identification and topic modeling.

We compare the performance of standard algorithms for collocation identification and topic modeling inference on a variety of corpora with different redundancy levels. We introduce synthetic corpora where we can control the level of redundancy. These synthetic corpora are derived from the Wall Street Journal (WSJ) standard corpus. The original WSJ corpus is naturally occurring and does not exhibit the copy and paste redundancy inherent to the EHR corpus. We artificially introduce redundancy by randomly sampling documents and repeating them until a controlled level of redundancy is achieved.

#### Collocation identification

We expect that in a redundant corpus, the word sequences (n-grams) which are copied often will be over-represented. Our objective is to establish whether the collocation algorithm will detect the same n-grams on a non-redundant corpus or on a version of the same corpus where parts of the documents have been copied.

Two implications of noise are possible. The first is false positive identification, *i.e.,* extracting collocations which are the result of mere chance. The second implication is loss of significant collocations due to noise (or because important collocations are out-ranked by less important ones).

We apply two mutual information collocation identification algorithms (PMI and TMI, see Methods section) to the *All Informative Notes* corpus (redundant) and to the *Last Informative Note* corpus (non-redundant). In this scenario, we control for vocabulary: only word types that appear in the smaller corpus (*Last Informative Note*) are considered for collocations. To measure the impact of redundancy on the extracted collocations, for each collocation, we count the number of patients whose notes contain this collocation. A collocation that is supported by evidence from less than three patients is likely to be a false positive signal due to the effect of redundancy (*i.e.*, most of the evidence supporting the collocation was created via a copy/paste process).

We observe that the lists of extracted collocations on these two corpora differ markedly (collocations were extracted with a threshold of 0.001 and 0.01 for TMI and PMI respectively). The PMI algorithm identified 15,814 collocations in the *All Informative Notes* corpus, and 2,527 in the *Last Informative Notes* corpus. When comparing the collocations extracted from the two corpora, we find that 36% of the collocations identified in the *All Informative Notes* corpus were supported by 3 patients or less, compared to only 6% in the *Last Informative Note* corpus. See Table 2. For example, a note replicated 5 times signed by “John Doe NP” (Nurse Practitioner) was enough to gain a high PMI of 10.2 for the “Doe NP” bigram (as “Doe” appears only in the presence of “NP”).

**Table 2:** Collocations found in redundant and non-redundant corpora

	**All informative (redundant)**	**Last informative (non-redundant)**
**Word Types**	81,928	40,774
**Words**	3,641,031	545,231
**Collocations**	15,814	2,527
**Collocations/Word**	0.004	0.004
**Avg. number of patients per collocation**	18.2	66
**% collocations that appear in notes of 3 patients or less**	36 %	1 %

Collocations were extracted using a stringent cutoff of 0.001 PMI.

The second type of error, loss of signal can also be observed. When comparing all collocations using the same TMI cutoff, the *All Informative Notes* corpus produces 3 times as many collocations as the *Last Informative Notes* corpus (see Table 3), but we find that only 54% of the collocations found in the non-redundant corpus are represented in the bigger list.

**Table 3:** Collocation detection results in the different corpora

	**All informative (redundant) – 8,557 notes**	**Last informative (non-redundant) - 1,247 notes**	**Reduced redundancy – 3,970 notes**
**Collocations (TMI/PMI)**	5,649/15,814	2,082/2,527	3,590/6,034
**Avg. number of patients per collocation (TMI/PMI)**	32/18	74/66	48/37
**% collocations that appear in notes of 3 patients or less (TMI/PMI)**	32%/36%	1.2%/1%	6.2%/5.8%

Another method for selecting the significant collocations is using a top-N cutoff instead of a PMI cutoff. Comparing the top 1,000 collocations with TMI for *All Informative Notes* and *Last Informative Notes,* we find a marked difference of 196 collocations.

To control for size, we repeated the same experiment on a standard large-scale corpus, the WSJ dataset, on which collocation identification algorithms have been heavily tested in the past (see Table 4).

**Table 4:** Comparison of extracted collocations

**Corpus name**	**Corpus type**	**Size of corpus# words / # distinct words**	**#extracted collocations (TMI / PMI)**	**Average #documents per collocation**
WSJ-400	Non-redundant	214 K / 19 K	551/565	20.2/19.9
WSJ-600	Non-redundant	309 K / 23.5 K	943/1,000	15.5/15.2
WSJ-1300	Non-redundant	680 K / 36 K	1,881/2,518	10.8/9.7
WSJs5	Synthetic Redundant	1.69 M (±42 K)/36 K	3,035±(63)/17,015±(950)	7.4±(0.11)/2.8±(0.09)

Comparison of extracted collocations on synthetic redundant corpora and non-redundant corpora (WSJ – X words / Y distinct words). Collocations were extracted using using True Mutual Information and Pointwise Mutual Information (with cutoffs of 0.001 and 0.01 respectively).

Consider a scenario where a corpus is fed twice or thrice in sequence to PMI (that is, every document occurs exactly twice or thrice), then the list of extracted collocations will be identical to that of the original corpus. This is expected based on the definition of PMI, and we confirm this prediction on WSJx2 and WSJx3 which produce exactly the same list of collocations as WSJ-1300 (WSJx2 is a corpus constructed by doubling every document in WSJ-1300).

We observe a different behavior on WSJs5 (see Table 4): in this corpus, original sentences from WSJ-1300 are sampled between 1 and 5 times in a uniform manner (this process was replicated 10 times to eliminate bias from the random sampling). On this synthetic corpus, we obtain a different list of collocations when using the PMI algorithm: 17,015(±950) instead of 2,737. The growth in number of extracted collocations is expected since WSJs5 is 2.5 times larger than WSJ-1300, but this growth is less than expected when comparing the trend (WSJ-400, WSJ-600, WSJ-1300) with a growth of (565, 1,000 and 2,737) extracted collocations.

On the other hand, the collocations acquired on the redundant WSJs5 corpus have much weaker support than those obtained on WSJ-1300 (they occur on average in 2.8±0.09 instead of 9.6 documents per collocation). The differences we observe in this experiment are caused by the fact that some sentences only are copied, in a variable number of times (some sentences occur once, some twice, and others 5 times). Thus, PMI (which does not simply reflect word frequencies in a corpus, but takes into account global patterns of co-occurrences, since it relies on the probability of seeing terms jointly and terms independently) does not behave similarly when fed with our different corpora.

In the case of this synthetic dataset, the newly acquired collocations are all due to the synthetic copy-paste process and are likely a false positive signal. One may ask, however, whether the fact that the sentences are repeated in EHR corpora reflects on their semantic importance from a clinical standpoint, and therefore, whether the collocations extracted from the full EHR corpus contain more clinically relevant collocations. This hypothesis is rejected by the comparison of the number of “patient-specific” collocations in the redundant corpus and non-redundant one: the collocations acquired on the redundant corpus cannot serve as general reusable terms in the domain, but rather correspond to patient-specific, accidental word co-occurrences such as (first-name last-name) pairs. In other words, the PMI algorithm does not behave as desired because of the observed redundancy. For example, through qualitative inspection of the extracted collocations, we observed that within the top-20 extracted collocations from the full EHR redundant corpus, 17 appear only in a single cluster of redundant documents (a large chain of notes of a single patient copied and pasted). The fact that redundancy never occurs across patients, but within same-patient notes only, seems to create unintended biases in the extracted collocations.

The results on the WSJ and its synthetic variants confirm our results on the EHR corpora: collocations extracted on a redundant corpus differ significantly from those extracted on a corpus of similar size without redundancy. Slightly weaker, though consistent, results were encountered when using an alternative algorithm for collocation identification on the EHR and WSJ corpora (TMI instead of PMI).

#### Topic modeling

The algorithm for topic modeling that we analyze, LDA, is a complex inference process which captures patterns of word co-occurrences within documents. To investigate the behavior of LDA on corpora with varying levels of redundancy, we rely on two standard evaluation criteria: log-likelihood fit on withheld data and the number of topics required in order to obtain the best fit on the withheld data. The higher the log-likelihood on withheld data, the more successful the topic model is at modeling the document structure of the input corpus. The number of topics is a free parameter of LDA – given two LDA models with the same log-likelihood on withheld data, the one with the lower number of topics has better explanatory power (fewer latent variables or topics are needed to explain the data).

**Figure 3:**
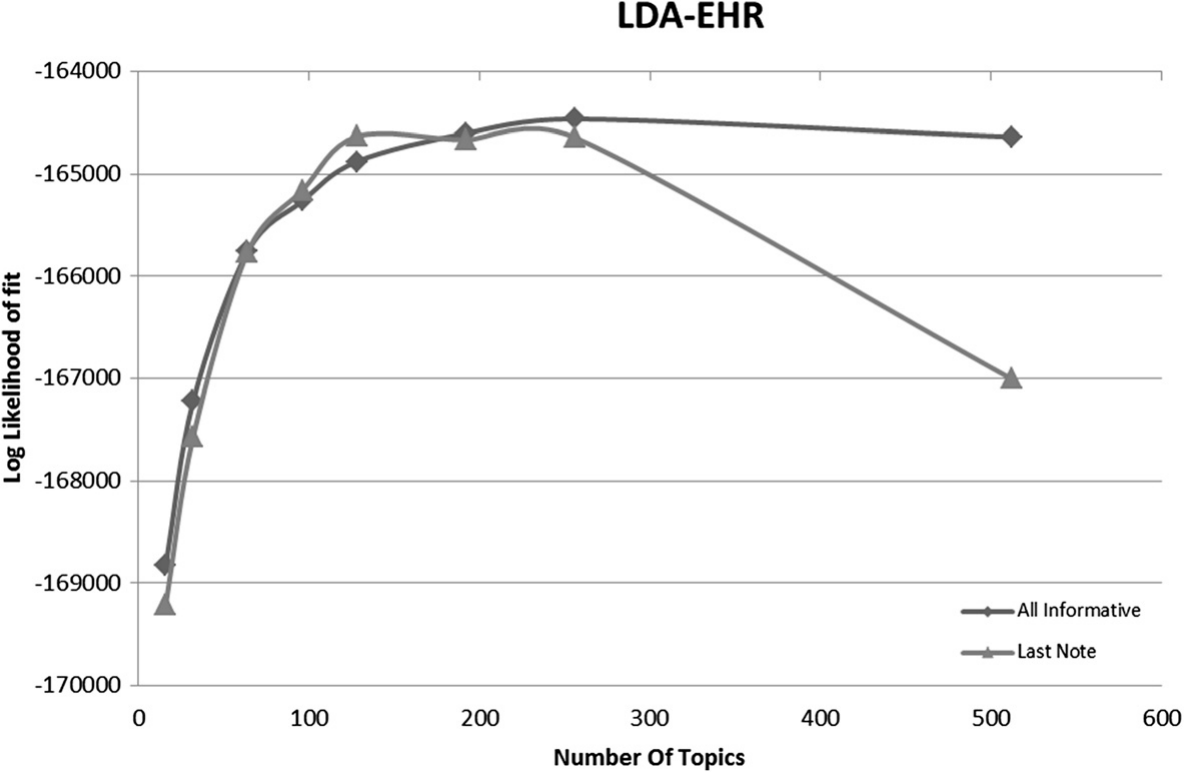
Model fit as function of number of topics on the EHR corpora.

We apply LDA to the same two EHR corpora (*All Informative Notes* and *Last Informative Note*) as in the collocation identification task, and obtained the results shown in Figure 3. The redundant corpus, though 6.9 times larger, produces the same fit as the non-redundant corpus (*Last Informative Note*).

**Figure 4:**
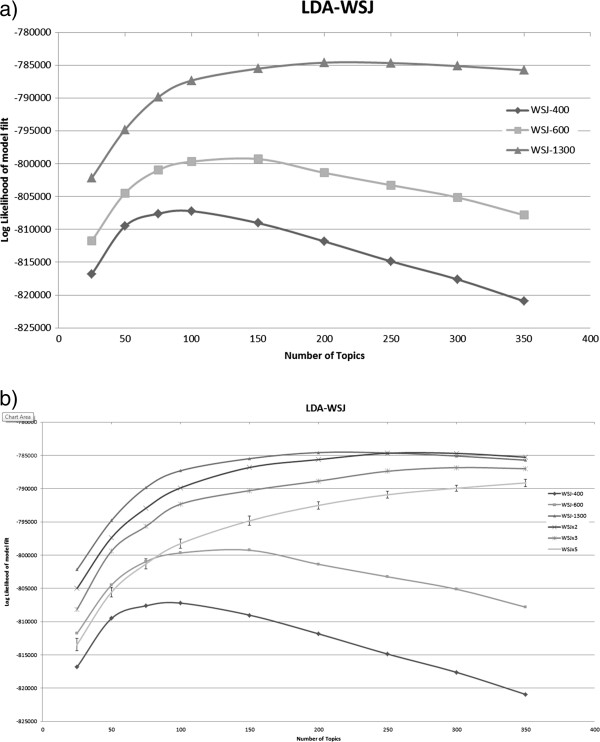
**Model fit as function of number of topics on the WSJ corpora.** In (**a**) we compare the effect of size on LDA, bigger corpora yield better fit. In (**b**) we examine the effect of redundancy: the doubled/trebled corpora reduce fit slightly while the noisier WSJs5 performs almost as badly as training on the smaller WSJ-600 corpus.

When applied to the synthetic WSJ corpora, we get a finer picture of the behavior of LDA under various corpora sizes and redundancy levels (Figure 4). The WSJ-400, WSJ-600 and WSJ-1300 corpora are non-redundant and have increasing size. We observe that the log-likelihood graphs for them have the same shape, with the larger corpora achieving higher log-likelihood, and the best fits obtained with topic numbers between 100 and 200 (Figure 4a). The behavior is different for the redundant corpora. WSJx2, WSJx3, and WSJs5 are all larger in size than WSJ-1300. We therefore would expect them to reach higher log-likelihood, but this does not occur. Instead, their log-likelihood graphs keep increasing as the number of topics increases, all the while remaining consistently inferior to the WSJ-1300 corpus, from which they are derived. The higher the redundancy level (twice, thrice or up-to-five times), the worse the fit.

Furthermore, when comparing WSJx3 and WSJs5 corpora (Figure 4b), which have roughly the same size, we note that the more redundant corpus (WSJs5 – 220% non uniform redundancy) has consistently lower fit to withheld data than WSJx3 (200% uniform redundancy). This confirms that redundancy hurts the performance of topic modeling, even when the size of the input corpus is controlled.

Even more striking, when examining the behavior of WSJs5 (with 3,300 documents sampled from 1,300 distinct documents) up to 100 topics, we observe it reaches the same fit as WSJ-600. That is, redundancy “confuses” the LDA algorithm twice: it performs worse than the original WSJ-1300 corpus although it contains the same documents, and the fit is the same as if the algorithm had roughly five times less documents (600 distinct documents from WSJ-600 vs. 1,300 distinct documents or 3,300 documents from WSJs5).

We have seen that for the naturally occurring WSJ corpus training on more data produces better fit to held out data (see Figure 4a). In contrast, we observe that the redundant *All Informative Notes* corpus, while 7 times larger than the non-redundant subset, does not increase log-likelihood fit to held out data.

To understand this discrepancy, we examine the topics obtained on the redundant corpora qualitatively. Topics are generated by LDA as ranked lists of words. Once a topic model is applied on a document, we can compute the topic assignment for each word in the document. We observe in the topics learned on the highly redundant corpora that the same word may be assigned to different topics in different copies of the same document. This lack of consistency explains the confusion and consequently low performance achieved by LDA on redundant corpora.

### Mitigation strategies for handling redundancy

Given a corpus with inherent redundancy, like the EHR corpus, the basic goal of redundancy mitigation is to choose the largest possible subset of the corpus with an upper bound on the amount of redundancy in that subset.

We compare two mitigation strategies to detect and handle redundancy in a corpus – a baseline relying on document metadata and one based on document content (which is applicable to the common case of anonymized corpora). We focus on the *All Informative Notes* corpus. The metadata-based baseline produces the *Last Informative Note* corpus. The content-based mitigation strategy, which relies on fingerprinting, can produce corpora with varying levels of redundancy. We report results for similarity thresholds of 0.20, 0.25 and 0.33. We expect that the lower the similarity threshold, the lower the actual redundancy level of the resulting corpus (in other words, we verify that our fingerprinting redundancy reduction algorithm effectively reduces redundancy).

#### Descriptive statistics of reduced corpora

Table 5 lists descriptive statistics of the corpora obtained with different methods. The input, Full EHR corpus, is the largest. As expected, the *Last Informative Note* corpus obtained through our metadata-based baseline is the smallest corpus. While redundancy is reduced, its size is also drastically decreased from the original corpus. As expected, the lower the maximum similarity threshold, the more stringent the criterion to include a document in the corpus, and thus, the smaller the resulting corpus.

**Table 5:** Descriptive statistics of the patient notes corpora

**Corpus**	**# Notes**	**# Words**	**# Concepts**
All Informative (input)	8,557	6,131,879	599,847
Last Informative Note (baseline)	1,247	435,387	44,145
Selective- Fingerprinting maximum similarity 0.33	4,524	3,614,409	337,034
Selective-Fingerprinting maximum similarity 0.25	3,970	3,283,558	302,159
Selective-Fingerprinting maximum similarity 0.20	3,645	3,061,854	278,644

All Informative, input corpus, the corpus obtained by the redundancy reduction baseline (*Last Informative Note*), and the corpora produced by the fingerprinting redundancy reduction strategy at different level.

Computation time for constructing a redundancy-reduced corpus at a given similarity threshold using the selective fingerprinting is 6 minutes (with an Intel Xeon CPU X5570 2.93 GHz).

To confirm that fingerprinting similarity effectively controls the redundancy level of the resulting corpora, we align a random sample of the notes included in the corpus for a sample of patients using different methods and different similarity cutoffs (see Table 6). The average amount of redundancy in removed note pairs is sampled as well. Redundancy is computed in the same way as in Section 2.1.1. We randomly sampled 2,000 same-patient pairs of notes and aligned them using Smith-Waterman alignment.

**Table 6:** Redundancy in same patient note pairs

**Corpus**	**Redundancy of in-corpus note pairs**	**Number of pairs in sample**
All Informative	29%	2,000
Selective- Fingerprinting maximum similarity 0.33	12.70%	380
Selective-Fingerprinting maximum similarity 0.25	9.80%	305
Selective-Fingerprinting maximum similarity 0.2	9.30%	263

Amount of redundancy in a random sample of 2,000 same-patient note pairs within the corpora using different similarity thresholds.

To investigate whether the corpora whose redundancy is reduced through our fingerprinting method are robust with respect to text mining methods, we focused on the following corpora: The inherently redundant *All Informative Notes* corpus, the baseline non-redundant corpus *Last Informative Notes* , and “*Reduced Redundancy Informative Notes*”, a corpus created by selective fingerprinting with maximum similarity of 25%. The *Reduced Redundancy Informative Notes* corpus contains 3,970 patient notes, 3.18 as many notes as the *Last Informative Notes* corpus while having same-patient redundancy of only 9.8% compared to 29% in the *All Informative Notes* Corpus.

#### Performance of text mining tasks on reduced corpora

For collocations detection, in *Reduced Redundancy Informative Notes*, 6,034 collocations were extracted, on average each collocation is supported by 37 distinct patients and collocations supported by 3 patients or less make 6% of the extracted collocation. We see a significant reduction in the number of collocations based on very few patients from 36% to 6% (Table 3).

**Figure 5:**
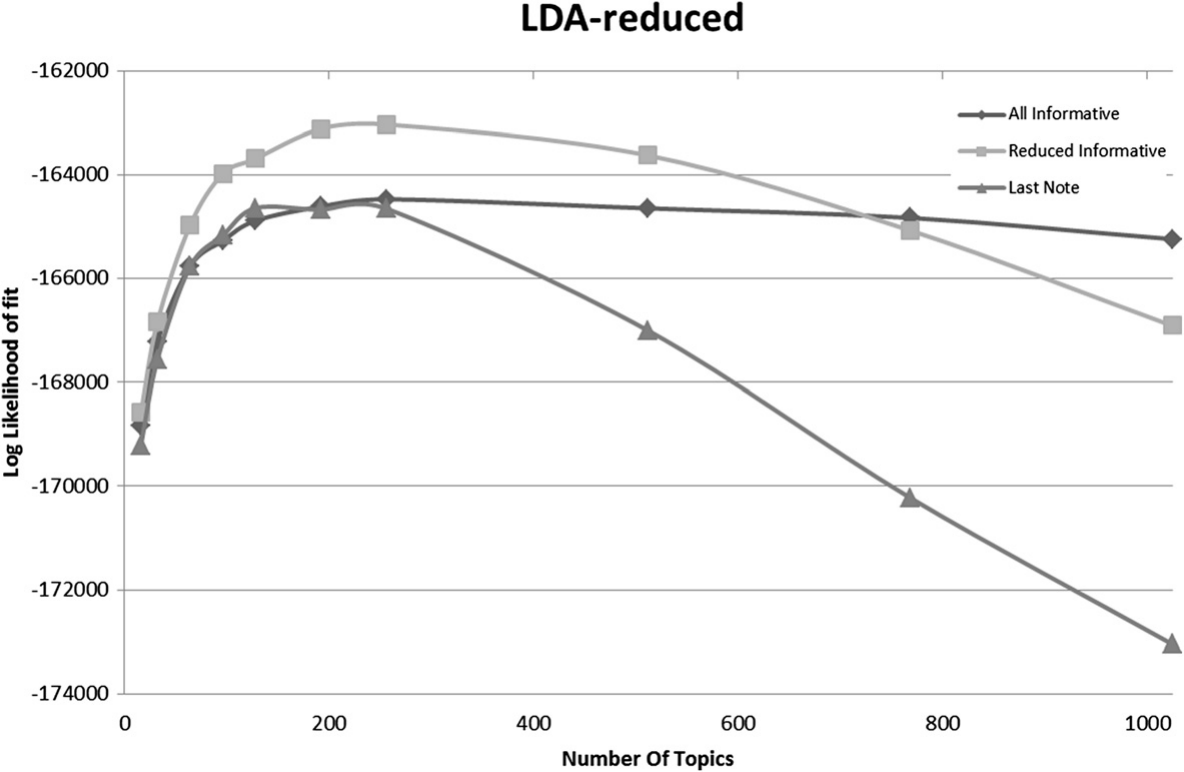
**Model fit as function of number of topics. Patient notes corpora, including the “*Reduced Informative*” corpus.**

For topic modeling, Figure 5 shows the log-likelihood fit on the EHR withheld dataset graphed against the number of topics for the LDA topic modeling for three corpora. We see that the significantly smaller *Last Informative Note* performs as well as *All Informative Notes* (8,557 notes vs. 1,247) while *Reduced Redundancy Informative Notes* (3,970 notes) outperforms both. As we showed in Figure 4a, we would expect a larger corpus to yield a better fit on the model: *All Informative Notes* is more than 7 times larger than *Last Informative*, still it yields the same fit on held out data. This is explained by the non-uniform redundancy of *All Informative* as shown in Figure 4b. In contrast, the *Reduced Redundancy Informative Notes* improves the fit compared to the non-redundant *Last Informative Notes* in the same manner as WSJ-1300 improves on WSJ-400 (a non-redundant corpus 3 times larger produces a better fit as expected). This healthy behavior strongly indicates that *Reduced Redundancy Informative Notes* indeed behaved as a non-redundant corpus with respect to the LDA algorithm.

## Conclusions

Training and improvement of NLP tools for Medical-Informatics tasks on public available data will continue growing as more EHRs are incorporated into health care givers worldwide. The nature of epidemiological research demands looking at cohorts of patients, such as our kidney patient notes. Such cohort studies require application of text mining and statistical learning methods for: collocation detection (such as PMI and TMI), Topic Modeling with LDA and methods for learning association between conditions, medication and more.

This paper identifies a characteristic of EHR text corpora: their inherent high level of redundancy, caused by the process of cut and paste involved in the creation and editing of patient notes by health providers. We empirically measure this level of redundancy on a large patient note corpus, and verify that such redundancy introduces unwanted bias when applying standard text mining algorithms. Existing text mining algorithms rely on statistical assumptions about the distribution of words and semantic concepts which are not verified on highly redundant corpora. We empirically measure the damage caused by redundancy on the tasks of collocation extraction and topic modeling through a series of controlled experiments. Preliminary qualitative inspection of the results suggests that idiosyncrasies of each patient (where the redundancy occurs) explain the observed bias.

This result indicates the need to examine the effect of redundancy on statistical learning methods before applying any other text mining algorithm to such data. In this paper, we focused on intrinsic, quantitative evaluations to assess the impact of redundancy on two text-mining techniques. Qualitative analysis as well as task-based evaluations are needed to get a full understanding of the role of redundancy in clinical notes on text-mining methods.

We presented a novel corpus subset construction method which efficiently limits the amount of redundancy in the created subset. Our method can produce corpora with different redundancy amounts quickly, without alignment of documents and without any prior knowledge of the documents. We confirmed that the parameter of our Selective Fingerprinting method is a good predictor of document alignment and can be used as the sole method for removing redundancy.

While methods such as our Selective Fingerprinting algorithm that extract a non-redundant / less-redundant subset of the corpus prevent bias, they still lead to lost information of the non-redundant parts of eliminated documents. An alternative route to text mining in the presence of high levels of redundancy consists of keeping all the existing redundant data, but designing redundancy immune statistical learning algorithms. This is a promising route of future research.

## Methods

### Datasets

#### EHR corpora

We collected a corpus of patient notes from the clinical data warehouse of the New York-Presbyterian Hospital. The study was approved by the Institutional Review Board (IRB-AAAD9071) and follows HIPAA (Health Insurance Portability and Accountability Act) privacy guidelines. The corpus is homogeneous in its content, as it comprises notes of patients with chronic kidney disease who rely for primary care on one of the institution’s clinic. Each patient record contains different note types, including consult notes from specialists (*e.g.,* nephrology and cardiology notes), admission notes and discharge summaries, as well as notes from primary providers, which synthesize all of the patient’s problems, medications, assessments and plans.

Notes contain the following metadata: unique patient identifier, date, and note type (*e.g.,* Primary-Provider). The content of the notes was pre-processed to identify document structure (section boundaries and section headers, lists and paragraph boundaries, and sentence boundaries), shallow syntactic structure (part-of-speech tagging with the GENIA tagger [46] and phrase chunking with the OpenNLP toolkit [47], and UMLS concept mentions with our in-house named-entity recognizer HealthTermFinder [48]). HealthTermFinder identifies named-entities mentions and maps them against semantic concepts in UMLS [49]. As such, it is possible to map lexical variants (*e.g., “myocardial infarction,” “myocardial infarct,” “MI,”* and *“heart attack”*) of the same semantic concept to a UMLS CUI (concept unique identifier).

There are 104 different note types in the corpus. Some are template based, such as radiology or lab reports, and others are less structured and contain mostly free text. We identified that note types: “*primary-provider*”, “*clinical-note*” and “*follow-up-note*” contain more information than other note types. Notes of these types were found to contain 37 CUIs on average in comparison to 26 on average for all other note types. We call notes of these 3 types “*Informative Notes*”.

In our experiments, we rely on different variants of the EHR corpus (see Table 7):

• The *All Notes* corpus is our full EHR corpus,

• The *All Informative Notes* corpus is a subset of All Notes, and contains only the notes of type “*primary-provider*”, “*clinical-note*” and “*follow-up-note*”.

• The *Last Informative Note* corpus is a subset of *All Informative Notes*, and contains only the most recent note for each patient.

**Table 7:** EHR corpora descriptive statistics

**Corpus**	**# Patients**	**# Notes**	**# Words / # Unique Words**	**# Concepts / # Unique Concepts**
All Notes	1,604	22,564	6,131,879 / 138,877	599,847 / 7,174
All Informative Notes	1,247	8,557	2,243,551 / 51,234	319,298 / 5,389
Last Informative Note	1,247	1,247	338,207 / 25,624	46,311 / 3,711

#### Synthetic WSJ redundant corpora

We construct synthetic corpora with a controllable level of redundancy to compare the behavior of the text mining methods on various levels of redundancy. The synthetic corpora are based on a sample of the Wall Street Journal corpus, a widely used corpus in the field on Natural Language Processing [50,51]. Table 8 provides descriptive statistics of the different WSJ-based corpora with which we experiment:

• The WSJ-1300 corpus contains a random sample of 1,300 documents from the Wall Street Journal corpus,

• The WSJ-400 corpus is a subset of WSJ-1300 of 400 documents,

• The WSJ-600 corpus is a subset of WSJ-1300 of 600 documents,

• The WSJx2 corpus is constructed from WSJ-1300 to simulate redundancy, where each document of WSJ-1300 appears twice in the corpus.

• The WSJx3 corpus is similar to the WSJx2 corpus, except it contains three copies of each document in the WSJ-1300 corpus.

• The WSJs5 corpus is sampled from WSJ-1300 corpus, where each document can appear between one and five times in the corpus, with a uniform probability of 0.2. Note that the WSJs5 corpus has roughly 2.5 times the size of WSJ-1300. The process was repeated 10 times to eliminate bias from the choice of documents repeated.

**Table 8:** Corpora Descriptive statistics

**Corpus**	**# Documents**	**# Words / # Unique Words**
WSJ-400	400	214 K / 19 K
WSJ-600	600	309 K / 23.5 K
WSJ-1300	1,300	680 K / 36 K
WSJx2	2,600	1.3 M words / 36 K
WSJx3	3,900	2.6 M words / 36 K
WSJs5	3,246(±40)	1.69 M (±42 K) words / 36 K

Synthetic corpora with various levels of redundancy , for WSJs5 we report averages and standard deviation based on 10 replications.

### Quantifying redundancy in the EHR corpus

#### Metric for assessing redundancy at the patient level

Given two notes, we computed redundancy for the pair by aligning the two notes. We applied the Smith-Waterman text alignment algorithm, a commonly used string alignment algorithm in bioinformatics [52]. For each pair, we can then compute the percentage of aligned tokens. Assessing redundancy through alignment is a more appropriate and more stringent method than counting simple token overlap as in a bag-of-word model. High percentage of alignment between two notes indicates not only that tokens are similar across the two notes, but that the sequences of tokens in the notes are also similar.

#### Metric for assessing redundancy at the corpus level

Given a corpus, a histogram of term frequencies is computed to examine whether the corpus follows Zipf’s law. According to Zipf’s law, terms frequencies have a long tail in their distribution: that is, very few terms occur frequently (typically function words and prominent domain words) while most terms occur only once or twice in the corpus overall. Terms can be either words or semantic concepts.

### Mutual information and topic modeling

Collocation identification was carried out on the different corpora using the Ngram Statistics Package [53], which provides an implementation for collocation detection using True Mutual Information (TMI) and Pointwise Mutual Information (PMI).

We compare LDA topic modeling based on log-likelihood fit to a test set and the number of topics required to obtain the best fit. This is similar to the approach used by Arnold *et al*. (2010) [28] and is accepted as a method for comparing LDA performance [54].

The topic models were learned using the Collapsed Gibbs Sampler provided in Mallet [55] with the recommended parameters and with hyper-parameter optimization as described in Wallach *et al.*[56]. The log-likelihood graphs were computed on withheld datasets. A non-redundant withheld dataset of 233 Informative notes was created for EHR corpus (all the notes from the same patients were removed from the redundant corpora to prevent contamination between corpora and the withheld dataset). For the WSJ corpora, a sample of 400 non-redundant documents was chosen as the withheld set.

### Mitigation strategies for handling redundancy

#### Metadata-based baseline

The metadata-based mitigation strategy leverages the note creation date, the note type and the patient identifier information and selects the last available note per patient in the corpus. This baseline ensures the production of a non-redundant corpus, as there is one note per patient only.

#### Fingerprinting algorithm

Detecting redundancy within the notes of a single patient is feasible using standard alignment methods borrowed from bioinformatics such as: Smith-Waterman [52], FastA [41] or Blast2seq [40]. However, some available EHR corpora are de-identified to protect patient privacy [57] and notes are not grouped by patients. Aligning all the note pairs in a corpus would be computationally prohibitive, even for optimized techniques (FastA, Blast2Seq).

Approximation techniques to make this problem tractable were developed in bioinformatics to search sequence databases and for plagiarism detection. In both fields, fingerprinting schemes are applied. In BLAST, short substrings are used as fingerprints, whose length is defined by biological significance. These substrings are also used for optimizing the alignment. For plagiarism detection, HaCohen-Kerner *et al.*[42] compare two fingerprinting methods: (i) Full fingerprinting – all substrings of length *n* of a string are used as fingerprints. This means that for a string of length *m*, *m-n+1* fingerprints will be used; and (ii) Selective Fingerprinting – non-overlapping substrings are chosen. This means that for a string of length *m*, *m/n* fingerprints will be used.

The parameter *n* is the granularity of the method, and its choice determines how stringent the comparison is. In order to compare two notes A and B, we compute the number of fingerprints shared by A and B. The level of similarity of B to A is defined as the ratio *(number of shared fingerprints) / (number of fingerprints in A).*

**Figure 6:**
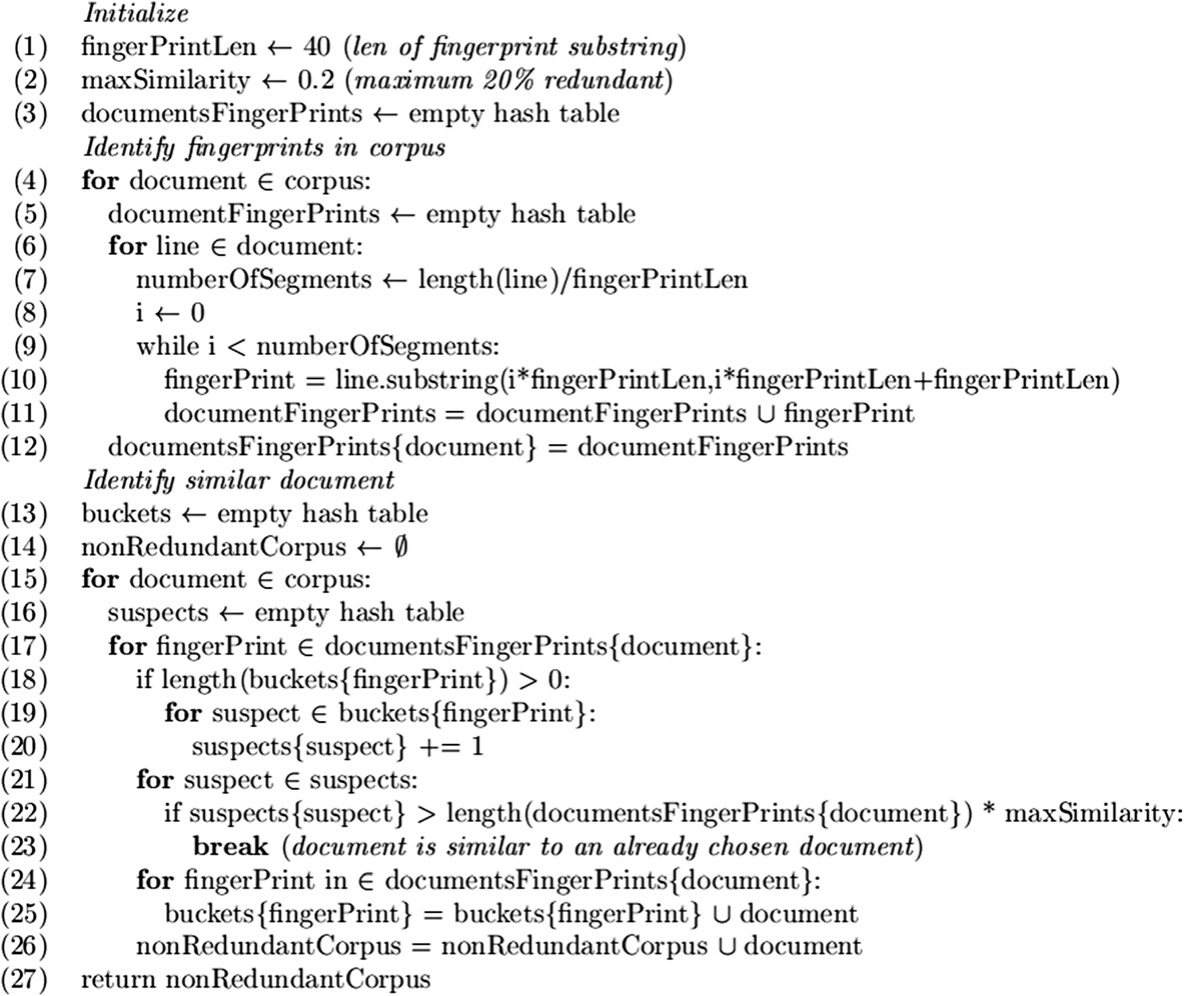
Pseudo Code of greedy controlled redundancy sub-corpus construction algorithm.

We use this fingerprinting similarity measure in the following redundancy reduction technique: fingerprints (non-overlapping substrings of length n) are extracted for each document line by line (*i.e.*, no fingerprint may span two lines). Documents are added one by one to the new corpus, a document sharing a proportion of fingerprints larger than the cutoff value with a document already in the corpus is not added. See Figure 6 for pseudo code of this algorithm. This method is a greedy approach similar to the online algorithm described in [58].

An implementation of our algorithm in Python together with all synthetic datasets is available at https://sourceforge.net/projects/corpusredundanc.

## Endnotes

^a^A Python implementation of our algorithm as well as all synthetic datasets are available at https://sourceforge.net/projects/corpusredundanc

## Competing interests

The authors declare that they have no competing interests.

## Authors’ contributions

RC participated in the study design, carried out the statistical analyses and wrote the paper. ME participated in study design and wrote the paper. NE participated in study design and wrote the paper. All authors read and approved the final manuscript.
